# Parrot beak nails: a Latin American case series^☆^

**DOI:** 10.1016/j.abd.2022.02.005

**Published:** 2022-11-07

**Authors:** Arias-Rodriguez Camilo, Beuth-Ruiz Santiago

**Affiliations:** a Universidad Pontificia Bolivariana, Medellín, Colombia; b Universidad de Antioquia, Medellín, Colombia

Dear Editor,

Parrot Beak Nail (PBN) deformity consists of a forward over the curvature of the distal nail plate, which gives the nail the appearance of this birds beak. Here in, we present three cases of PBN dystrophy, we describe a new association, and include a brief literature review.

A 75-year-old man with a medical history of leprosy diagnosed 20 years ago, who received complete treatment, came to the dermatologic clinic due to stasis dermatitis. On physical examination, as a coincidental finding, several and significant sequelae were found. He presented PBN with involvement of all his fingernails, chromonychia and onycholysis on diverse nails, contracture of the fingers sparing the thumb, and atrophy of the thenar and hypothenar muscles. He denied any symptoms, or history of trauma, and had not noticed this deformity (Fig. 1).

**Fig. 1:**
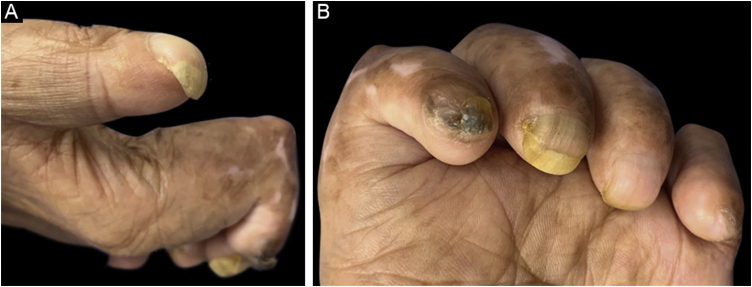
Images of the 1st patient. (A) First fingernail of the left hand showing PBN deformity. (B) All the fingernails were involved, with associated contracture of all the fingers and atrophy of hand muscles. Second fingernail shows focal plate thickening and brown chromonychia, third fingernail shows onycholysis and yellow chromonychia, and longitudinal melanonychia of third and fourth fingernails is noticed.

The second case was a 54-year-old woman with a diagnosis of rosacea, who consulted due to a recent flare. As a coincidental finding, PBN deformity of the fifth left fingernail was recognized. She had a history of left-hand trauma with a knife when she was eight years old (Fig. 2).

**Fig. 2:**
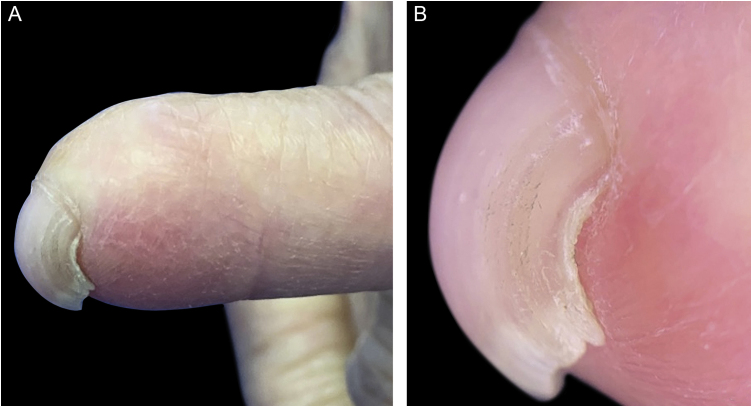
Images of the 2nd patient. (A) PBN deformity of the fifth left fingernail. (B) Dermoscopy enhances the visualization of the characteristic nail plate over curvature.

The third patient was an 80-year-old man with a personal history of hypertension, diabetes, and cerebrovascular disease, hospitalized due to gait disturbance. During his evaluation, his thumb and second right finger were partially amputated, and the third fingernail had a PBN deformity. He stated these changes were caused by the explosion of homemade fireworks at the age of thirteen (Fig. 3).

**Fig. 3:**
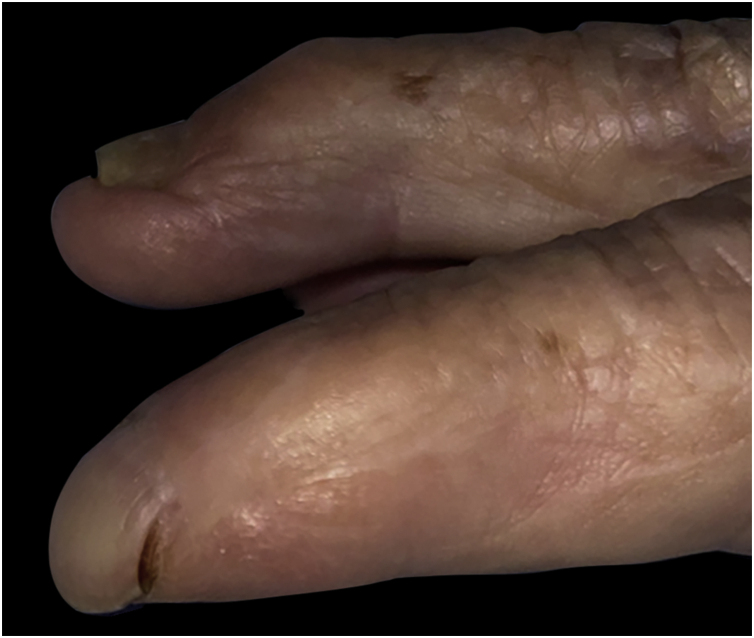
Image of the 3rd patient. PBN deformity secondary to trauma of the second fingernail, with associated pulp atrophy.

Kandil was the first one that described this deformity back in 1971, naming it due to its resemblance to a parrot beak. He reported an idiopathic over the curvature of two fingernails in a 38-year-old woman.^1^ Chen and Cohen reported a prevalence of 2.1% of 436 patients who consulted a dermatology clinic. Marie et al. observed this condition in 2.5% of 80 healthy individuals. Other case series have shown that 31% of patients with systemic sclerosis can present this nail dystrophy.^2^, ^3^ This deformity has been found in 11 to 89-year-old patients, without sex predominance. We found nine articles reporting this clinical finding, with a total of 78 patients (Table 1).

**Table 1:** Reported cases of parrot beak nail deformity.

Authors*	n	Year	Age	Sex	Compromised nail	Nail associations	Systemic associations
Kandil	1	1971	38	Female	Third and fourth fingernails	None	Idiopathic
Kurokawa et al.	2	1993	11, 17	Female	Toenails	None	Congenital soft tissue abnormalities and bony hypoplasia
Payne-James et al.	8	2007	24 to 40	Female	Fingernails	Perniosis, bolstering of the proximal nail folds and loss of cuticles	Chronic cocaine abuse
Tunc et al.	11	2007	40 to 68	Unknown	Not stated	None	Rheumatoid arthritis and systemic sclerosis
Desai et al.	1	2011	55	Female	Fingernails	None	Idiopathic
Chang et al.	2	2016	Not stated	Male and female	Not stated	None	Systemic sclerosis and mixed connective tissue disease
Marie et al.	42	2017	Not stated	Unknown	Fingernails	None	Idiopathic
Chen et al.	10	2017	63 to 89	Male	Toenails	Onycholysis, subungual haemorrhage	Idiopathic, systemic disorders
Forouzan et al.	1	2021	86	Male	Fourth toenail	Distal onycholysis	Pulmonary disease, dementia, hypothyroidism, prostate, and non-melanoma skin cancer
Arias-Rodriguez et al.	3	2022	54, 75, 80	Male and female	Fingernails	None	Leprosy and trauma

* References of the articles can be found in supplementary file.

Its etiology remains unknown. PBN has been associated with several conditions, including collagen vascular diseases, such as systemic lupus erythematosus and systemic sclerosis, where it may be the first finding and has been related to disease activity. Additional associations include cocaine abuse, and trauma, among other disorders.^4^, ^5^ The most accepted theory proposes that it is the result of an abnormal phospholipid distribution, which causes hydrophobic interactions between different zones of the nail plate.^1^ Authors who support this theory claim the over curvature seen in PBN can be temporarily corrected after submerging the affected nail in water for some minutes, since it would overcome those hydrophobic interactions.^2^ An injury could be the main cause, as in the present study’s second and third cases. It would generate a chronic imbalance of growth and alter the content of hydrophobic phospholipids, leading to a pronounced longitudinal curvature.

Other theories include chronic vasoconstrictive ischemia as a key factor, based on a case series of eight women with chronic cocaine abuse who developed this deformity.^6^ Furthemore, PBN is common in patients with systemic sclerosis, when associated with vascular impairment.^7^, ^8^ It could also be secondary to bone or soft tissue disorders, which may be congenital.

Repeated trauma is in certain cases the main cause. PBN dystrophy is found in patients with peripheral neuropathy who are prone to unnoticed nail bed traumatisms. Digital amputation and tight surgical closure in fingertip surgery are causes of pulp atrophy and extensive scarring, which could lead to a hooked-nail deformity that resembles PBN. Some surgical techniques have been described for its prevention: nail relocation, and hypodermic needles for tension-free closure, among others.^1^

When it is associated with chronic cocaine abuse, a triad of PBN, perniosis, and finger pulp atrophy has been reported.^6^ Other comorbidities mentioned in the literature, whether they are coincidental or unassociated findings, include bony dystrophy, coronary artery disease, lymphoplasmacytic sclerosing pancreatitis, multiple system atrophy, digit deformity (hammer toe, overlying the fifth toe), and soft tissue hypoplasia.

Neuropathy-associated cases were previously reported by Forouzan et al., who described a patient unaware of his toenail elongation, due to an androgen deprivation therapy-induced peripheral neuropathy.^2^ Other neuropathy-associated cases described by Chen et al. include diabetes and spinal stenosis-induced neuropathy. The present study’s first patient had leprosy neuropathy; an association not described to the date in the revisited literature.

Clinically, PBN can involve one or several fingernails or toenails, however, it is more common in fingernails. When toenails are affected, there is usually an association with congenital abnormalities, neuropathies, or systemic conditions. On the other hand, in individuals with fingernail involvement, most cases are idiopathic. Other nail apparatus components, such as proximal nail plate, nail bed, nail matrix, and soft tissue, remain unaffected; however, PBN can coexist with other nail dystrophies such as onychocryptosis, onychogryphosis, onycholysis, erythronychia, subungual hemorrhage, and onychotillomania.^9^

Usually, this deformity is asymptomatic, therefore subreported, but if the deformed nail grows towards the palmar or plantar surfaces, it can damage acral skin, with the risk of superinfection. Diagnosis is clinical, a semiological aid consists in soaking the nail in water for 30 minutes, which would temporarily correct the deformity.^2^

Among differential diagnoses, one can distinguish clubbing from PBN, because of the normal curvature of the nail plate in the former one. Pachyonychia congenita patients can present with curved nails, but they are also thick and brown or yellowish. Onychogryphosis is mostly due to ill-fitting shoes. Other disorders such as congenital curvature of the fourth toenail may also resemble PBN.

Periodic nail cutting should be recommended to prevent continuous growth. PBN can be cured with an onychectomy and matricectomy, which may benefit patients with symptomatic or recurrent cases.^10^

## Financial support

None declared.

## Authors' contributions

Camilo Arias-Rodriguez and Santiago Beuth-Ruiz contributed to the present study concept and design, data collection, writing and final approval of the manuscript.

## Conflicts of interest

None declared.
