# Supplementary material for: Parrot beak nails: a Latin American case series
Source: An Bras Dermatol. 2022 Nov 7;98(1):96–9. doi: 10.1016/j.abd.2022.02.005 (PMC9837638; doi:10.1016/j.abd.2022.02.005)
Supplement: Supplementary file 1 [file mmc1.pdf]

References of articles cited on table 1.

1. Kandil E. Parrot beak nails. J Med Liban. 1971;24:433-6.

2. Kurokawa M, Isshiki N, Inoue K. A new treatment for parrot beak deformity of the toe. Plast Reconstr Surg. 1994;93:558-60.

3. Payne-James JJ, Munro MH, Payne CMER. Pseudosclerodermatous triad of perniosis, pulp atrophy and 'parrot-beaked' clawing of the nails--a newly recognized syndrome of chronic crack cocaine use. J Forensic Leg Med. 2007;14:65-71.

4. Tunc SE, Ertam I, Pirildar T, Turk T, Ozturk M, Doganavsargil E. Nail changes in connective tissue diseases: Do nail changes provide clues for the diagnosis? J Eur Acad Dermatology Venereol. 2007;21:497–503.

5. Desai T, Magdum A, Patel T, Loghdey S. Parrot-beak nails. Clin Exp Dermatol. 2011;36:208–9.

6. Chang P, Tello GA, Cohen Sabban EN, Anzueto E. Manifestaciones del aparato ungueal en las enfermedades del colágeno: reporte de 43 casos. Dermatologia Cosmet Medica y Quir. 2016;14:270–80.

7. Marie I, Gremain V, Nassermadji K, Richard L, Joly P, Menard JF, et al. Nail involvement in systemic sclerosis. J Am Acad Dermatol. 2017;76:1115–23.

8. Chen SX, Cohen PR. Parrot Beak Nails Revisited: Case Series and Comprehensive Review. Dermatol Ther (Heidelb). 2018;8:147–55.

9. Forouzan P, Cohen PR. Parrot Beak Nail: Case Report and Review of Parrot Beak Nail Dystrophy. Cureus. 2021;13:1–7.
